# Changes in the Sodium Content of New Zealand Processed Foods: 2003–2013

**DOI:** 10.3390/nu7064054

**Published:** 2015-05-27

**Authors:** David Monro, Cliona Ni Mhurchu, Yannan Jiang, Delvina Gorton, Helen Eyles

**Affiliations:** 1 Heart Foundation of New Zealand, PO Box 17160, Greenlane, Auckand 1546, New Zealand; E-Mails: davem@heartfoundation.org.nz (D.M.); info@heartfoundation.org.nz (D.G.); 2 National Institute for Health Innovation, The University of Auckland, Private Bag 92019, Auckland 1142, New Zealand; E-Mails: c.nimhurchu@auckland.ac.nz (C.N.M.); y.jiang@auckland.ac.nz (Y.J.); 3 Epidemiology and Biostatistics, The University of Auckland, Private Bag 92019, Auckland 1142, New Zealand

**Keywords:** sodium, salt, processed food, packaged food, food analysis, New Zealand

## Abstract

Decreasing population sodium intake has been identified as a “best buy” for reducing non-communicable disease. The aim of this study was to explore 10-year changes in the sodium content of New Zealand processed foods. Nutrient data for nine key food groups were collected in supermarkets in 2003 (*n* = 323) and 2013 (*n* = 885). Mean (SD) and median (min, max) sodium content were calculated by food group, year and label type (private/branded). Paired *t-*tests explored changes in sodium content for all products available for sale in both years (matched; *n* = 182). The mean (SD) sodium content of all foods was 436 (263) mg (100 g)^−1^ in 2003 and 433 (304) mg (100 g)^−1^ in 2013, with no significant difference in matched products over time (mean (SD) difference, −56 (122) mg (100 g)^−1^, 12%; *p* = 0.22). The largest percentage reductions in sodium (for matched products) were observed for Breakfast Cereals (28%; −123 (125) mg (100 g)^−1^), Canned Spaghetti (15%; −76 (111) mg (100 g)^−1^) and Bread (14%; −68 (69) mg (100 g)^−1^). The reduction in sodium was greater for matched private *vs.* branded foods (−69 *vs.* −50 mg (100 g)^−1^, both *p* < 0.001). There has been modest progress with sodium reduction in some New Zealand food categories over the past 10 years. A renewed focus across the whole food supply is needed if New Zealand is to meet its global commitment to reducing population sodium intake.

## 1. Introduction

Salt is the leading source of sodium in the human diet and has been used for many generations as a key ingredient in cooking and food manufacturing. However, high salt intakes are linked to high blood pressure, the leading risk factor for early death globally [1]. Salt intakes in most high income countries far exceed the World Health Organization (WHO) guideline for reducing blood pressure and risk of cardiovascular disease, stroke, and coronary heart disease in adults (<5 g salt or <2000 mg sodium day^−1^ [2]); UK, 8.1 g salt day^−1^ (3200 mg sodium; 2011) [3]; US, 8.3 g salt day^−1^ (3330 mg sodium; 2008 [4]); and New Zealand, 8.4 g salt day^−1^ (3373 mg sodium; 2012).

The majority of dietary energy and nutrients (~75%) consumed in high income countries come from processed foods [5]. As such, healthier reformulation of processed foods has gained increasing support in the past decade. Reduction of sodium in packaged foods was identified at the 2011 United Nations (UN) high level meeting as a “best buy” for reducing non-communicable diseases (NCDs) and is likely to be cost effective [6,7,8]. Moreover, reduction of population salt intakes by 30% relative to 2010 levels (by 2025) is one of nine global targets agreed upon by UN committed member states (including New Zealand) [9].

Nonetheless, sodium reduction efforts globally have been mixed, with a number of governments and health agencies creating programmes to work in partnership with the food industry to encourage sodium reduction, yet only nine countries have legislation to improve population salt intakes [10]. Of the countries which have national sodium reduction strategies, manufacturer targets for reduction of salt in processed foods are a mainstay of the plan; in the UK such targets have led to a 7% fall in the salt content of processed foods [11] and contributed to a reduction in salt intakes from 9.5 g day^−1^ in 2001 to 8.1 g day^−1^ in 2011 [3]. However, there has been very little robust monitoring of changes in the salt content of packaged foods internationally. 

In New Zealand there is no government-led national sodium reduction strategy, yet some food companies have created their own internal salt reduction programmes. Furthermore, the Heart Foundation of New Zealand has been running the Tick Programme which encourages reformulation and healthier consumer food choices, since 1991. The Tick is a manufacturer-funded initiative in Australia and New Zealand where products that meet certain nutrient criteria are eligible to carry a Tick logo to indicate a healthier choice within category [12]. In 2010 the Heart Foundation of New Zealand also established HeartSAFE to support food companies to meet voluntary sodium reduction guidelines [13]. In order to produce the greatest population health impacts the HeartSAFE programme is particularly focused on reducing sodium in low cost, high volume foods, including private label (retailer own) products. The aim of this study was to explore 10-year (2003 to 2013) changes in the sodium content of key packaged foods in New Zealand. Changes in sodium content by food group and category, and by label (private or branded) were also explored.

## 2. Experimental Section

### 2.1. Data Sources, Food Groups and Categories

In 2003, data on the sodium content of New Zealand food products were collected from the four major supermarkets in Dunedin, New Zealand [14]. Sodium values were collected directly from the Nutrition Information Panels (NIP) on product packages and entered into Microsoft Excel. Data were collected for nine food categories (Table 1) selected on the following criteria:

(1) Were major contributors to population sodium intakes [15]
(2) Included a wide range of sodium contents across the food category; a wide range of sodium contents indicates the potential or feasibility for producing lower sodium products, *i.e.*, sodium reduction
(3) Displayed a NIP (a new Food Standards Code came into effect in 2002 requiring almost all packaged food to display a NIP) [16]. In 2003, some food products did not yet meet these requirements as companies were still working towards the requirements of the new Code (e.g., sausages)

Corresponding data for 2013 were obtained from the Nutritrack database, a branded food composition database managed by the National Institute for Health Innovation at The University of Auckland [17]. Nutritrack data are collected directly from the NIPs of all packaged food products available for sale at major supermarkets in Auckland. A bespoke smartphone application was used to collect data where product photos were taken and nutrition information entered into an on-line database (NutriWeb). Auckland is New Zealand’s largest city with approximately one third of the country’s supermarkets (~1200 stores). Supermarkets chosen for data collection in 2013 represented the largest stores of the biggest retail brands of the two main supermarket retailers in New Zealand (Foodstuffs (54% grocery market share) and Progressive Enterprises (38% market share [18])). The nine food groups were further broken down into smaller categories for analysis. Food groups and categories included are shown in Table 1.

### 2.2. Data Cleaning and Matching

Only one package size for each food product was included in the analysis. This was to ensure that averages were not skewed by products with multiple pack sizes. Where the sodium content differed between different pack sizes the value for the most common pack size with the greatest shelf presence was used.

Value range checks were carried out on the sodium values for all products and checked against source data and photographs where necessary and possible. Some exclusions were made to ensure the types of products included for 2003 and 2013 were consistent. For example, in 2003 rolled oats and “heat and serve” porridge style cereals were not included in the Breakfast Cereal food group because they contained very little sodium and were not considered a focus for reformulation. Therefore, they were also excluded from the 2013 Breakfast Cereal data set. Similarly, wraps and pita breads were excluded from the 2013 data set because these types of products were not available in 2003.

**Table 1:** Sodium content of key packaged food products available for sale in New Zealand supermarkerts in 2003 and 2013.

		Sodium content 2003 (mg 100 g^−1^)							Sodium content 2013 (mg 100 g^−1^)						
		*N*	% total foods *	Mean	SD	Median	Min	Max	*N*	% total foods *	Mean	SD	Median	Min	Max
Bread	All	45	14%	472	61	450	350	600	104	12%	410	68	410	380	630
	White	17	5%	516	52	515	450	600	23	3%	433	62	410	346	630
	Whole Meal/Grain	28	9%	445	51	450	350	540	81	9%	403	68	410	1	530
Breakfast cereals^$^	All	109	34%	348	275	289	6	933	176	20%	215	183	205	1	780
	Childrens’	34	11%	574	286	577	6	933	42	5%	358	229	378	2	780
	Museli	22	7%	111	125	61	14	479	47	5%	64	81	43	4	479
	Other	44	14%	303	216	286	10	714	74	8%	211	133	215	1	520
	Wheat Biscuits & Bites	9	3%	297	76	280	205	437	13	1%	317	54	300	235	410
Butter, Margarine & Dairy Blends	All	50	15%	474	135	400	360	772	58	7%	421	139	360	1	700
	Butter	7	2%	480	0	480	480	480	11	1%	579	47	600	480	600
	Dairy Blends	6	2%	467	123	400	390	700	9	1%	394	34	400	360	470
	Margarine	37	11%	474	150	380	360	772	38	4%	382	140	360	1	700
Canned Corned Beef	All	10	3%	770	265	750	463	1369	11	1%	718	164	750	460	920
Canned Salmon	All	9	3%	347	195	425	80	600	32	4%	364	134	369	59	580
Canned Spaghetti	All	9	3%	474	127	460	370	800	13	1%	370	125	390	1	505
Canned Vegetables	All	39	12%	241	174	270	0	780	116	13%	200	137	185	0	583
	Asparagus	7	2%	289	23	280	280	340	7	1%	238	62	240	120	310
	Baked Beans	9	3%	445	194	365	270	780	22	2%	384	124	415	0	583
	Beetroot	4	1%	273	157	335	40	380	12	1%	234	67	237	70	330
	Creamed Corn	7	2%	167	55	150	140	290	8	1%	194	65	165	130	300
	Tomatoes	7	2%	90	89	60	0	190	56	6%	120	96	128	0	350
	Whole Corn Kernels	5	2%	94	52	110	5	140	11	1%	185	90	162	2	340
Cheese ^^^	All	15	5%	661	56	630	590	800	59	7%	673	61	670	315	760
Crackers	All	37	11%	637	369	610	8	1390	316	36%	604	367	594	0	1810
Total		323		436	263	430	0	1390	885		433	304	400	0	1810

^$^ Other cereals included light flakes and fruit, brans, and all other cereals; ^Cheese included only plain hard cheeses, e.g., Edam, Colby as these were the only types of cheeses collected in 2003; * Percentage of total foods based on proportion of total products in analysis, e.g., 45 breads of 323 products = 14%.

Manual data matching was carried out in Excel for products available for sale in both 2003 and 2013. Where changes had been made to product names, authors (DM, HE) used their knowledge of the New Zealand grocery sector to match products as appropriate.

### 2.3. Statistical Analysis

For all products in the dataset, mean (SD) and median (min, max) sodium content was calculated for each year overall, by food group and category, and by label type (private or branded). Means for private label and branded sub-categories were calculated for food groups, but not for smaller food categories due to insufficient numbers. 

Significance testing was not undertaken for all products available for sale (unpaired data) due to the large difference in numbers and types of products available in each food group in 2003 compared with 2013. Two-sided paired *t*-tests were undertaken on all products that were available for sale in both years to explore changes in sodium content over 10 year time period, *i.e.*, matched data. These data were normally distributed and a significance level of 5% was used. Significance testing was not undertaken by food group due to small product numbers and differences between means and medians indicating these data were skewed. No adjustment was made for multiple comparisons. However, the number of tests was considered in interpreting the findings. 

## 3. Results

The final dataset included 323 products from 2003 and 885 products from 2013. One hundred and eighty two products were available for sale in both 2003 and 2013. 

### 3.1. Sodium Content of Food Groups and Categories in 2003 and 2013

The mean (SD) and, median (range) sodium content overall for each year and for food groups and categories in 2003 and 2013 are shown in Table 1. 

The mean (SD) sodium content of all foods was 436 (263) mg (100 g)^−1^ in 2003 and 433 (304) mg (100 g)^−1^ in 2013. Food categoriess with the highest mean (SD) sodium content per 100g for both 2003 and 2013 were Canned Corned Beef (770 (265) mg (100 g)^−1^ and 718 (164) mg (100 g)^−1^, respectively), Cheese (661 (56) mg (100 g)^−1^ and 673 (61) mg (100 g)^−1^, respectively), and Crackers (637 (369) mg (100 g)^−1^ and 604 mg (367) (100 g)^−1^, respectively). Food categories with the lowest mean sodium content in both years were Muesli (111 (125) mg (100 g)^−1^ in 2003 and 64 (81) mg (100 g)^−1^ in 2013), Canned Tomatoes (90 (89) mg (100 g)^−1^ *vs.* 120 (96) mg (100 g)^−1^, respectively), and Canned Whole Corn Kernels (94 (52) mg (100 g)^−1^ *vs.* 175 (162) mg (100 g)^−1^, respectively) (Table 1).

### 3.2. Change in Sodium Content of Matched Products Available for Sale in Both 2003 and 2013

**Figure 1:**
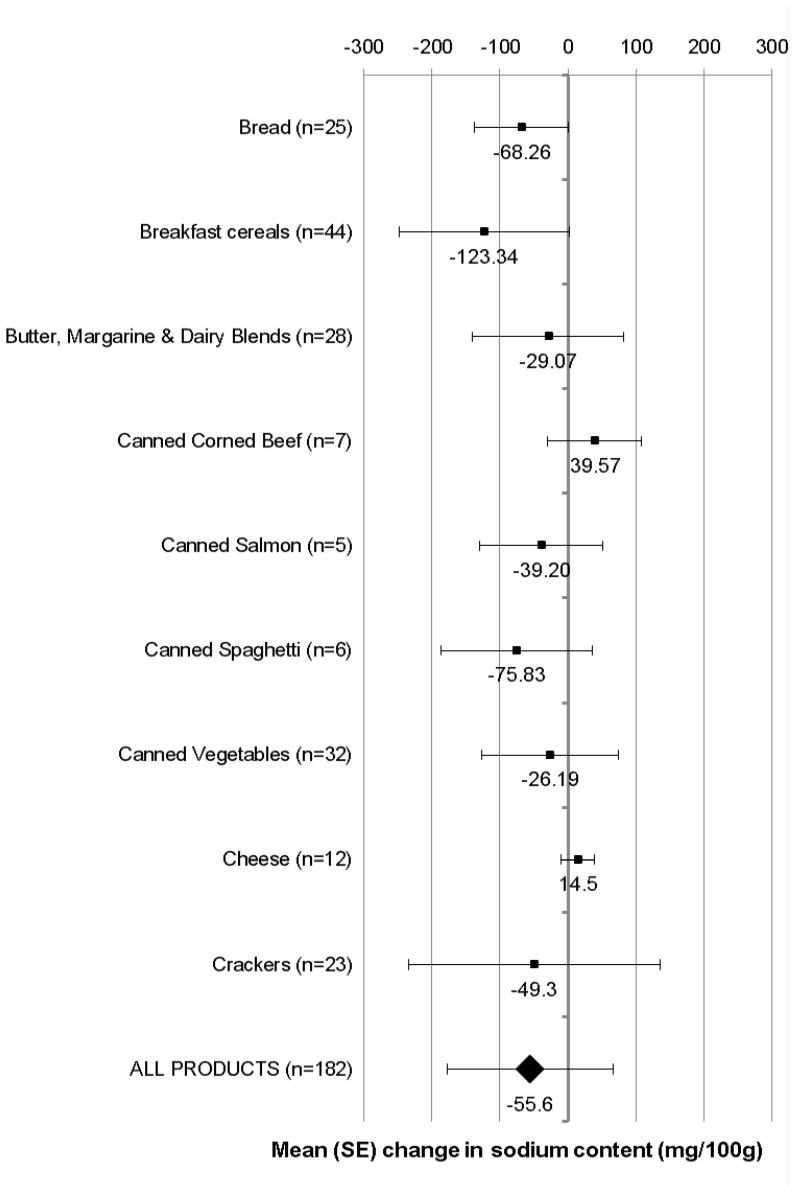
Mean difference in the sodium content of matched processed food products available for sale in both 2003 and 2013 (*n* = 182).

The mean (SD) sodium content of matched products available for sale (*n* = 182) in 2003 was 454 (257) mg(100 g)^−1^ and in 2013 was 399 (232) mg (100 g)^−1^ (Supplementary data). The overall mean (SD) difference in sodium content was −56 (122) mg (100 g)^−1^ or 12% (*p* = 0.22; Figure 1). In seven of nine food groups assessed reductions in mean sodium content were evident (Figure 1), although there were too few products within food groups to undertake significance testing. The largest percentage reductions in sodium were observed for Breakfast Cereals (28%; mean (SD difference, −123 (125) mg (100 g)^−1^), Canned Spaghetti (15%; −76 (111) mg (100 g)^−1^) and Bread (14%; −68 (69) mg (100 g)^−1^). For two food groups there was an increase in mean sodium content (mean (SD) difference: Canned Corned Beef (40 (91) mg (100 g)^−1^ or 6%, and Cheese (15 (24) mg (100 g)^−1^ or 2%).

### 3.3. Sodium Content of Private Label and Branded Products in 2003 and 2013

For both years and for the majority of food groups assessed, the mean sodium content of private label products was higher than that of branded products (Table 2). The largest differences in mean sodium between branded and private label products in any year were: Canned Corned Beef in 2013 (645 *vs.* 910 mg (100 g)^−1^, respectively), Butter and Margarine in 2003 (421 *vs.* 608 mg (100 g)^−1^, respectively), Canned Salmon in 2003 (297 *vs.* 445 mg (100 g)^−1^), and Canned Spaghetti in 2013 (345 *vs.* 453 mg (100 g)^−1^, respectively) (Table 2).

Overall, there were statistically significant reductions in the sodium content of branded (*n* = 132) and private label (*n* = 52) matched products available for sale in both years (mean (SD) reduction: −50 (118) mg (100 g)^−1^ (*p* < 0.001) and −69 (133) mg (100 g)^−1^ (*p* < 0.001), respectively). There were too few products available to undertake paired/matched analyses by food group or category.

## 4. Discussion

The aim of this study was to explore 10-year (2003 to 2013) changes in the sodium content of New Zealand processed foods. The number of overall products available for sale each year in selected food groups was 323 in 2003 and 885 in 2013. 

The mean (SD) sodium content of all products available for sale was similar in 2003 and 2013 (436 (263) mg (100 g)^−1^ *vs.* 433 (304) mg (100 g)^−1^, respectively). No significant difference was observed in the overall sodium content of matched products (available for sale in both years; *n* = 182; mean (SD) difference −56 (122) mg (100 g)^−1^, or 12%; *p* = 0.22). Too few products were available to undertake significance testing of matched products by food group. However, food groups with the largest percentage reductions were Breakfast cereals (28%), Canned Spaghetti (15%) and Bread (14%). Increases in sodium were observed for 2/9 food groups assessed (Canned corned beef and Cheese). Overall, the sodium content of matched branded (*n* = 132) and private label (*n* = 52) products reduced significantly over the ten year time period (mean (SD) reductions: −50 (118) mg (100 g)^−1^ (*p* < 0.001) in 2003 and −69 (133) mg (100 g)^−1^ (*p* < 0.001) in 2013).

These data are novel in that little data exists nationally on the sodium content of the processed food supply over longer periods of time, including for branded *vs.* private label foods, and across a wide range of food categories. However, the following limitations must be considered in reading the study findings. First, there were major changes in the food supply between 2003 and 2013 which impacted on the current analysis. For example, the availability of different types of food products has changed; the current analyses included 37 types of crackers in 2003 *vs.* 316 in 2013, and some products (such as sachets of flavoured oats) were not available in 2003. Changes in the number of products in a category may impact the overall mean sodium content of food products as categories with more products will provide a greater percentage contribution to the overall concentration of sodium in the total food supply. For example, crackers which have a high sodium content (637 mg (100 g)^−1^ in 2003 and 604 mg (100 g)^−1^ in 2013) comprised 11% of all products measured in 2003 and 36% of products in 2013. Inclusion of new products such as sachets of flavoured oats may also impact the overall mean sodium concentration, but would not have affected matched/paired analyses. Thus new products coming onto the market may increase the overall sodium concentration and off-set reformulation undertaken in foods such as Bread and Breakfast Cereals. Nonetheless, this reflects actual availability of sodium to consumers.

**Table 2:** Sodium content of key packaged food products available for sale in New Zealand supermarkets in 2003 and/or 2013, by brand or private label.

		Sodium Content 2003 (mg (100 g)^−1^)						Sodium Content 2013 (mg (100 g)^−1^)					
		*N*	Mean	SD	Median	Min	Max	*N*	Mean	SD	Median	Min	Max
**Bread**	**Branded**	29	463	59	450	350	560	85	408	73	410	1	630
	**Private Label**	16	488	65	490	366	600	19	416	34	410	366	503
**Breakfast cereals ^$^**	**Branded**	73	320	247	289	6	920	146	201	167	191	1	725
	**Private Label**	36	406	320	294	7	933	30	279	237	280	3	780
**Butter, margarine, and dairy blends**	**Branded**	36	421	89	380	360	700	52	414	136	360	1	700
	**Private Label**	14	608	142	575	377	772	6	486	157	475	339	680
**Canned corned beef**	**Branded**	9	772	281	750	463	1369	8	645	128	637	460	820
	**Private Label**	1	750	.	750	750	750	3	910	9	905	905	920
**Canned salmon**	**Branded**	6	297	227	273	80	600	24	373	124	375	76	580
	**Private Label**	3	445	39	468	400	468	8	337	165	367	59	538
**Canned spaghetti**	**Branded**	3	420	40	420	380	460	10	345	130	385	1	455
	**Private Label**	6	502	151	460	370	800	3	453	58	465	390	505
**Canned vegetables**	**Branded**	25	223	166	190	0	740	79	208	146	185	0	583
	**Private Label**	14	273	190	280	10	780	37	185	113	168	5	440
**Cheese ^**	**Branded**	7	669	72	670	590	800	42	672	69	685	315	760
	**Private Label**	8	653	41	630	620	710	17	676	34	668	620	740
**Crackers**	**Branded**	33	632	384	610	8	1390	277	605	378	600	0	1810
	**Private Label**	4	678	238	690	410	920	39	598	284	553	100	1370
**ALL PRODUCTS**	**Branded**	221	421	269	400	0	1390	723	436	312	400	0	1810
	**Private Label**	102	468	247	475	7	933	162	417	265	400	3	1370

^$^ Other cereals included light flakes and fruit, brans, and all other cereals; **^^^** Cheese included only plain hard cheeses, e.g., Edam, Colby as these were the only types of cheeses collected in 2003.

Other limitations of the current analyses include that they may not represent the entire New Zealand food supply and are not weighted by sales; inclusion of sales weighted data gives a better indication of where best to reformulate to achieve the largest health gain [19]. Furthermore, a more precise picture of the trend in sodium levels would have been achieved should data have been available for each of the years between 2003 and 2013, and collection made from the same city and supermarkets to keep datasets more consistent. The collection of data in 2013 was more comprehensive and took place in a larger city meaning a larger overall data set for 2013 and a greater number of products within certain categories. However, some of this increase in products would likely have been due to natural increases in product variety in the food supply during the 10 year period of data coverage. Finally, our analyses do not include processed foods purchased for consumption outside the home including fast food and other restaurants. Food away from home is a significant contributor to nutrient intakes and comprises approximately one-quarter to one third of household food purchases by expenditure in New Zealand [20,21].

Reductions observed in matched food products in New Zealand over the past 10 years suggest reformulation efforts are likely due, at least in part, to manufacturer own internal sodium reduction programmes and support for sodium reduction from the Heart Foundation in the form of HeartSAFE [13] and the Tick Programme [22]. HeartSAFE for example has had sodium reduction guidelines in place for bread, breakfast cereals, and some processed meats (ham, bacon and sausages, but excluding canned corned beef) since 2010, and the Tick programme was estimated to have removed 33 tonnes of salt from the New Zealand food supply in the one year period between July 1998 and June 1999 [22].

However, these analyses illustrate that while progress with sodium reduction has been made in some important New Zealand food groups over the past 10 years, little progress has been made in the sodium density of the overall New Zealand food supply. As such, Government leadership of a national sodium reduction strategy including widespread action across the whole food supply and larger reductions in leading food sources such as bread will be key to achieving the UN (committed) 30% relative reduction [23] in the sodium intakes by 2025.

In addition to widespread reformulation efforts these analyses indicate where sodium reduction needs particular focus. Specific food groups and categories where there has been little change in the overall sodium concentration over the past 10 years (or even increases in sodium) were: Wheat Biscuits and Bites, Butter, Canned Salmon, Canned Tomatoes and Corn, and Cheese (Table 1). Moreover, food groups where little reformulation has taken place (according to matched analyses) were Canned Corned Beef and Cheese (Figure 1). The wide ranges observed (Table 1) across food groups and categories illustrate potential for sodium reduction, even in food groups where reductions have already been made. 

Despite progress with reformulation in some food groups, the finding of no significant change in the average sodium content across the New Zealand processed food supply over the past 10 years is in contrast to what has been achieved via The United Kingdom’s (UK) salt reduction programme, which was led by the Food Standards Agency for approximately 10 years and is now being overseen by the Food Responsibility Deal [24]. The UK programme is recognised as one of the most successful salt reduction programmes globally and resulted in a 7% reduction (−26 mg (100 g)^−1^; *p* ≤ 0.001) in the sodium concentration of all processed foods between 2006 and 2011 indicating reformulation across the wider food supply [11]. In addition, a corresponding 7% reduction (−23 mg (100 g)^−1^; *p* < 0.001) was observed for matched/paired products. The reduction in sodium in UK processed foods was accompanied by a decrease in population dietary sodium intake, as assessed by 24-h urinary sodium excretion over a similar time period (1.4 g of salt (560 mg sodium) per day from 2001 to in 2011) [3]. Although the current New Zealand analysis included only nine food groups, compared with the UK analysis of the whole food supply, the reductions achieved in the UK for Bread and Breakfast Cereals were larger than the current findings for New Zealand (20% *vs.* 14% and 50% *vs.* 27%, respectively) [11].

In Australia the Food and Health Dialogue was launched by Federal Government in 2009 with voluntary targets for 10 categories of processed food. The targets for Bread, Ready-to-eat breakfast cereals and Processed meat were scheduled to be achieved by December 2013. Similar to the current findings for New Zealand, reductions were observed in the mean sodium content of matched Breads (New Zealand, 487 to 419 mg (100 g)^−1^ or 14%; Australia, 454 to 415 mg (100 g)^−1^ or 9%) and Breakfast Cereals (448 to 325 mg (100 g)^−1^ or 27% and 316 to 237 mg (100 g)^−1^ or 25%, respectively) [25]. Processed meats were not assessed in the current analyses, but reductions were observed over four years in Australia (2009 to 2013; 1215 to 1114 mg (100 g)^−1^ or 8%).

The UK and Australia successes indicate that a government-led national salt reduction strategy should be now be considered for New Zealand. Such a strategy should include targets for manufacturers, but also support as per the UK programme including a public awareness campaign of the importance of salt and health, and improved labelling (a new Health Star Rating front of pack labelling system has just been launched in New Zealand). 

Further research should explore trends over time in the sodium content of the entire New Zealand food supply, ideally weighted by sales [19]. Key categories omitted from this analysis but important in terms of sodium intakes in New Zealand include Sauces, Soups, and Processed meats (particularly sausages, bacon and ham; processed meats in New Zealand have been shown to have a higher sodium content compared with the United Kingdom and Australia [26]).

## 5. Conclusions

There has been modest progress with sodium reduction in some New Zealand food categories over the past 10 years. However, increased action across the whole food supply and a greater focus on food categories which have shown minimal change or have increased since 2003 (Wheat Biscuits and Bites, Butter, Canned Salmon, Canned Tomatoes and Corn, Cheese, and Corned Beef) is now required. This is essential if New Zealand is to meet its commitment to a 30% relative reduction in population sodium intake by 2025 [23].

## Supplementary Files

Supplementary File 1
