# Supplementary material for: Changes in the Sodium Content of New Zealand Processed Foods: 2003–2013
Source: Nutrients. 2015 May 27;7(6):4054–67. doi: 10.3390/nu7064054 (PMC4488772; doi:10.3390/nu7064054)
Supplement: Supplementary File 1 [file nutrients-07-04054-s001.docx]

**Supplementary Material**

**Table S1**. Sodium content of matched packaged food products available for sale in New Zealand supermarkets in both 2003 and 2013 (*n* = 182).

|  |  | **Sodium content 2003 (mg (100g)^−1^)** | | | | | | **Sodium content 2013 (mg (100g)^−1^)** | | | | | |
| --- | --- | --- | --- | --- | --- | --- | --- | --- | --- | --- | --- | --- | --- |
| **Bread** |  | *N* | Mean | SD | Median | Min | Max | *N* | Mean | SD | Median | Min | Max |
|  | All | 25 | 487 | 68 | 505 | 366 | 600 | 25 | 419 | 29 | 410 | 380 | 503 |
|  | White | 8 | 556 | 31 | 548 | 505 | 600 | 8 | 424 | 39 | 410 | 380 | 503 |
|  | Whole/grain | 17 | 455 | 54 | 450 | 366 | 540 | 17 | 417 | 23 | 415 | 380 | 456 |
| **Breakfast  cereals** | All | 44 | 448 | 284 | 477 | 6 | 920 | 44 | 325 | 218 | 353 | 3 | 780 |
|  | Childrens’ | 22 | 603 | 277 | 650 | 6 | 920 | 22 | 415 | 222 | 440 | 3 | 780 |
|  | Muesli | 6 | 179 | 192 | 93 | 14 | 479 | 6 | 98 | 187 | 19 | 7 | 479 |
|  | Others ^$^ | 12 | 346 | 209 | 365 | 10 | 605 | 12 | 279 | 168 | 277 | 18 | 520 |
|  | Wheat Biscuits & Bites | 4 | 307 | 88 | 275 | 240 | 437 | 4 | 306 | 53 | 283 | 275 | 385 |
| **Butter, Margarine & Dairy Blends** | All | 28 | 438 | 110 | 380 | 360 | 770 | 28 | 409 | 109 | 360 | 330 | 680 |
|  | Butter | 3 | 480 | 0 | 480 | 480 | 480 | 3 | 600 | 0 | 600 | 600 | 600 |
|  | Dairy Blends | 2 | 400 | 0 | 400 | 400 | 400 | 2 | 400 | 0 | 400 | 400 | 400 |
|  | Margarine | 23 | 436 | 121 | 380 | 360 | 770 | 23 | 385 | 95 | 360 | 330 | 680 |
| **Canned Corned Beef** | All | 7 | 645 | 138 | 630 | 463 | 820 | 7 | 685 | 169 | 750 | 460 | 905 |
| **Canned Salmon** | All | 5 | 341 | 225 | 425 | 90 | 600 | 5 | 301 | 197 | 403 | 76 | 468 |
| **Canned Spaghetti** | All | 6 | 497 | 152 | 460 | 380 | 800 | 6 | 421 | 67 | 423 | 320 | 505 |

**Table S1**. *Cont.*

|  |  | **Sodium content 2003 (mg (100g)^−1^)** | | | | | | **Sodium content 2013 (mg (100g)^−1^)** | | | | | |
| --- | --- | --- | --- | --- | --- | --- | --- | --- | --- | --- | --- | --- | --- |
| **Canned Vegetables** | All | 32 | 236 | 179 | 190 | 5 | 780 | 32 | 210 | 141 | 185 | 2 | 583 |
|  | Asparagus | 6 | 280 | 0 | 280 | 280 | 280 | 6 | 258 | 37 | 258 | 205 | 310 |
|  | Baked Beans | 7 | 464 | 213 | 365 | 270 | 780 | 7 | 401 | 121 | 410 | 220 | 583 |
|  | Beetroot | 3 | 250 | 184 | 330 | 40 | 380 | 3 | 198 | 112 | 250 | 70 | 275 |
|  | Creamed Corn | 6 | 147 | 8 | 145 | 140 | 160 | 6 | 162 | 33 | 160 | 130 | 222 |
|  | Tomatoes | 6 | 105 | 87 | 113 | 10 | 190 | 6 | 62 | 61 | 55 | 2 | 125 |
|  | Whole Corn Kernels | 4 | 91 | 59 | 110 | 5 | 140 | 4 | 105 | 70 | 128 | 2 | 162 |
| **Plain Hard Cheeses ^^^** | All | 12 | 659 | 39 | 650 | 620 | 710 | 12 | 674 | 31 | 678 | 620 | 710 |
| **Crackers** | All | 23 | 604 | 408 | 570 | 8 | 1390 | 23 | 555 | 360 | 498 | 7 | 1320 |
| **Total Products** |  | 182 | 454 | 257 | 450 | 5 | 1390 | 182 | 399 | 232 | 400 | 2 | 1320 |

^$^ Other cereals included light flakes and fruit, brans, and all other cereals, collected in 2003; ^Cheese included only plain hard cheeses, e.g., Edam, Colby as these were the only types of cheeses collected in 2003.
